# Prevalence of previously unrecognized peripheral arterial disease in patients undergoing coronary angiography

**DOI:** 10.1097/MD.0000000000011519

**Published:** 2018-07-20

**Authors:** Akram Saleh, Hanna Makhamreh, Tareq Qoussoos, Izzat Alawwa, Moath Alsmady, Zaid A. Salah, Ali Shakhatreh, Lewa Alhazaymeh, Mohammed Jabber

**Affiliations:** a Cardiology Department, Internal Medicine Department; b Internal Medicine Department, the University of Jordan Hospital, Amman; c Sixth-year Medical Student, Faculty of Medicine, the University of Jordan, Jordan.

**Keywords:** ankle-brachial index, asymptomatic, atherosclerosis, coronary artery disease, epidemiology, peripheral arterial disease

## Abstract

Coronary artery disease (CAD) and peripheral arterial disease (PAD) are serious manifestations of systemic atherosclerosis. A considerable proportion of patients with CAD have associated PAD; however, many are asymptomatic and this condition remains underdiagnosed. Little is known about the prevalence and clinical implication of PAD in patients undergoing coronary angiography in the Middle East with no history of the disease.

To study the prevalence of previously unrecognized PAD of the lower limbs in patients undergoing coronary angiography, and to determine the correlation with CAD.

This is a prospective study conducted at a university tertiary referral hospital. A total of 2120 patients referred for coronary angiography without a prior diagnosis of PAD, between January 1, 2014 and December 31, 2014, were included. Patients were evaluated through detailed medical history taking, a questionnaire survey to assess symptoms and functional status, ankle-brachial index (ABI) measurement, and coronary angiography. PAD was considered present if the ABI was <0.90 in either leg.

In all patients, the prevalence of previously unrecognized PAD was 12.8%. There was no significant difference between men and women (13.4% vs 11.7%, *P* = .485). Abnormal angiographic results were seen in 82% (1739 of 2120). The prevalence of PAD was 14.7% in patients with abnormal coronary angiographic result, significantly higher than that in patients with normal results (4.5%, *P* < .0001). The prevalence of abnormal angiographic results among patients with and without PAD was 96% and 80%, respectively (*P* = .001). Factors independently related to PAD were age (odds ratio [OR] 1.081, 95% confidence interval [CI] 1.053–1.109; *P* < .001), hypertension (OR 3.122, 95% CI: 1.474–5.678; *P* < .004), diabetes (OR 1.827, 95% CI: 0.975–2.171; *P* = .04), smoking (OR 1.301, 95% CI: 0.725–2.076; *P* < .001), previous coronary artery bypass grafting (OR 2.939, 95% CI: 1.385–5.219; *P* = .004), previous cerebrovascular accident (OR 3.212, 95% CI: 1.872–9.658; *P* = .003), left main CAD (OR 9.535, 95% CI: 3.978–20.230; *P* = .002), and multivessel CAD (OR 1.869, 95% CI: 1.018–2.798; *P* = .03). Patients with CAD and PAD were associated with a higher prevalence of multivessel CAD (58.2% vs 42.6%, *P* < .005) and left main disease (3% vs 0.3%, *P* < .0001).

The prevalence of undiagnosed PAD in patients undergoing coronary angiography was 12.8% (14.7% in patients with CAD) and associated with a higher incidence of cardiovascular risk factors, multivessel disease, and left main disease. The high prevalence of PAD in patients with CAD confirms the importance of active screening for PAD by using ABI. Routine determination of ABI in the clinical evaluation of all patients with CAD may help identify high-risk patients.

## 1 Introduction

Atherosclerosis is a progressive and diffuse pathological process that can simultaneously affect the coronary and peripheral arteries.^[1]^ The prevalence of peripheral arterial disease (PAD) increases with age, ranging from 1% to 3% in the fourth decade to >20% in the eighth decade.^[2,3]^ PAD is associated with an increased incidence of multivessel and obstructive coronary artery disease (CAD), and is a risk factor for cardiovascular events.^[4–7]^ In most cases, PAD is asymptomatic, and because the ankle-brachial index (ABI) is not routinely measured, PAD is largely underdiagnosed and therefore undertreated.^[8]^ Early detection of PAD in patients with CAD is essential for preventing the local progression of the disease and for an effective secondary prevention of future coronary events.^[9,10]^ ABI measurement is a noninvasive and sensitive method for evaluating atherosclerosis in the lower limbs, with values <0.9 indicating the presence of PAD.^[11]^ The ABI has been shown to have good sensitivity and specificity for PAD, as documented by direct comparisons with angiographic results, and is considered an independent predictor of coronary morbidity and mortality.^[12–17]^ Currently, little is known about the actual incidence and clinical implications of asymptomatic PAD in patients undergoing coronary angiography in the Middle East. We conducted this study to assess the prevalence of previously unrecognized PAD among patients with suspected ischemic heart disease undergoing coronary angiography, and to determine the relationship among PAD, severity of coronary angiographic stenosis, and major cardiovascular risk factors in one Middle Eastern population.

## 2 Methods

### 2.1 Study population

In this prospective study, we recruited participants with an unknown PAD status from among patients undergoing coronary angiography at the University of Jordan Hospital between January 2014 and January 2015. The ethics committee of the University of Jordan Hospital approved the study protocol. All participants signed an informed written consent form. We allowed the patients 15 minutes of rest before starting the study protocol. We obtained the characteristics of the studied participants, including demographics; atherosclerotic risk factors such as diabetes mellitus, hypertension, hyperlipidemia, and smoking; familial history; past medical history; and functional status by filling a detailed structured questionnaire administered by trained study personnel. Atherosclerotic risk factors were defined according to standard definitions.^[18–20]^ Patients were considered to have hypertension if they had an elevated systolic blood pressure >140 mm Hg and/or a diastolic blood pressure >90 mm Hg on several occasions during the hospital stay, were diagnosed as having hypertension, or were prescribed with antihypertensive medications by a treating physician. Diabetes mellitus was defined according to the standard criteria set by the American Diabetes Association, as follows: fasting serum glucose >126 mg/dL, 2-hour glucose level >200 mg/dL or glycosylated hemoglobin value >6.5%. Diabetes mellitus was also diagnosed in patients who had unequivocal hyperglycemia, classical symptoms of diabetes mellitus (polyuria, polydipsia, and unexplained weight loss), and casual plasma glucose level >200 mg/dL, and those with a prior diagnosis of diabetes mellitus or who were prescribed with antidiabetic medications by a treating physician. Patients who were cigarette smokers at enrollment were considered current smokers. Patients who never smoked and were past smokers who had quit at least 1 month before enrollment were considered nonsmokers. Patients were considered to have hypercholesterolemia if they had a past diagnosis by a treating physician, were prescribed with lipid-lowering agents, or were found to have a serum total cholesterol level of >240 mg/dL during the index admission. Body mass index was calculated according to the standard formula (body weight [kg]/height [m^2^]). Obesity was defined as a body mass index of >30 kg/m^2^. A family history of premature cerebrovascular disease was defined as myocardial infarction, coronary revascularization, or sudden death before age 55 years in the father or any other male first-degree relative, or before age 65 years in the mother or any other female first-degree relative.

Patients were excluded if they had a preexisting confirmed diagnosis of PAD, deformity in the upper and lower limbs, significant lower limb edema, unwillingness or inability to provide informed consent, and ABI >1.3 (noncompressible arteries).

### 2.2 Measurement of ABPI

After resting in the supine and head in midline position for 15 minutes, ABI was measured using the WatchBP Office ABI instrument. Blood pressure cuffs are tied to all 4 limbs and systolic pressure of all the limbs is measured at the same time and the ABI is calculated for each side using the higher systolic pressure of the 2 arms. Patients were identified as having PAD if their ABI was <0.9 in either leg. On the basis of the ABPI findings, we divided the participants into a normal group with no PAD (PAD−) and an abnormal group with PAD (PAD+).

All studied patients underwent coronary artery angiography via the femoral/radial approach with a 6-Fr catheter. All coronary segments were interpreted visually by an experienced cardiologist blinded to participant details. We defined significant stenosis on coronary angiography as >50% stenosis of an epicardial coronary artery. To assess the extent and severity of the CAD cases, we investigated whether multivessel and left main coronary lesions were involved or not. On the basis of the angiographic findings, we divided the participants into a normal group with no significant coronary stenosis (CAD−) and an abnormal group with significant coronary stenosis (CAD+). We compared the results of the questionnaire and abnormal coronary angiographic findings between the PAD+ and PAD− groups. After considering the clinical manifestations, we classified CAD cases into the following: stable ischemic heart disease (SIHD) and acute coronary syndrome (ACS).

### 2.3 Statistical analysis

We used SPSS software (version 20; Chicago, IL) for data description and analysis. Continuous variables are reported as mean ± standard deviation, unless otherwise indicated. Continuous variables were compared using unpaired *t* test. We used the Chi-square test to compare the categorical variables. A multivariate logistic regression model was used to identify the independent risk factors for PAD. The model included prespecified risk factors of old age, hypertension, diabetes mellitus, hyperlipidemia, current smoking, previous coronary artery bypass grafting (CABG), and a history of stroke. We analyzed the results and expressed them as odds ratios (ORs) for the comparison of risk with 95% confidence intervals (CIs). We considered *P* < .05 as indicating statistical significance.

## 3 Results

**Figure 1:**
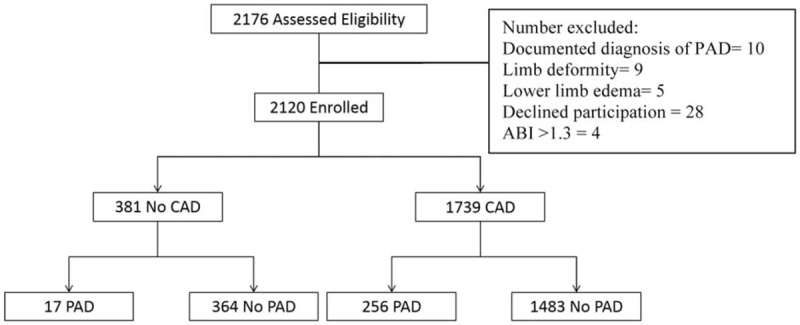
Participant flow chart. ABI = ankle-brachial index, CAD = coronary artery disease, PAD = peripheral arterial disease.

**Figure 2:**
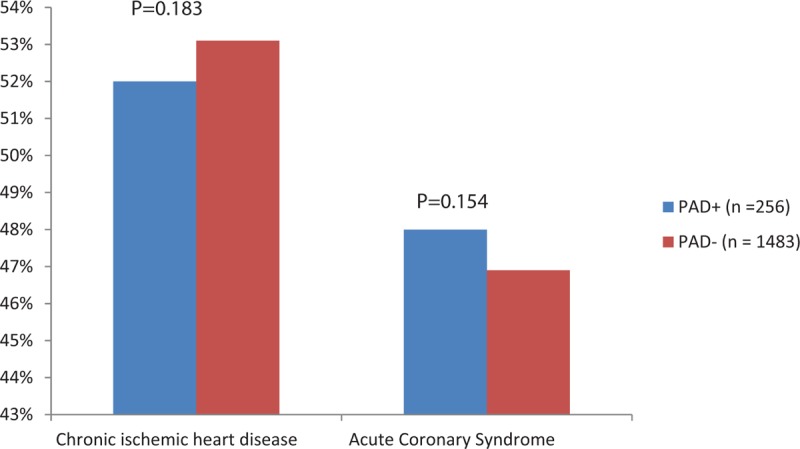
Clinical presentations. PAD = peripheral artery disease.

**Figure 3:**
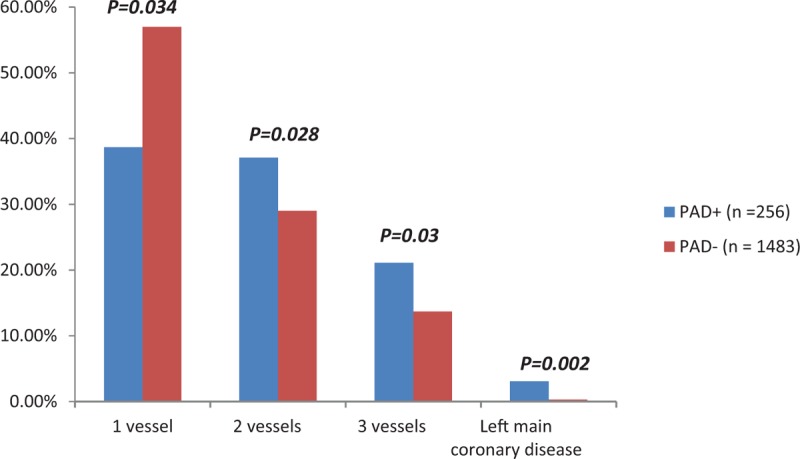
Severity of coronary artery disease. PAD = peripheral artery disease.

We reviewed all of the 2176 patients managed between January 2014 and January 2015 for the study; however, we subsequently excluded 56 (2.3%) patients based on the exclusion criteria. Therefore, we enrolled 2120 patients, including 1483 (70%) diagnosed as having CAD alone, 256 (12%) with CAD and PAD, and 381 (18%) without CAD (Fig. 1). The prevalence of PAD was 14.7% in patients with CAD, which was significantly higher than that in patients with normal coronaries (4.5%, *P* < .001). Table 1 shows the baseline demographic and clinical characteristics of the patients with CAD. Patients with PAD were significantly older, and had higher rates of hypertension, dyslipidemia, diabetes mellitus, current smoking, history of CABG, history of stroke, and transient ischemic attacks than patients without PAD. We did not find significant differences in the prevalence of PAD between male (15%) and female (12.5%) patients. The prevalence of CAD among PAD+ and PAD− patients was 96% and 80%, respectively (*P* = .001). There were no significant differences in the prevalence of PAD according to the patients’ clinical presentation of either SIHD or ACS (Fig. 2). Patients with PAD tended to more often present with multivessel CAD (57.4% vs 42.6%, *P* < .001) and left main disease (3.1% vs 0.3%, *P* = .003) than those who did not have PAD (Fig. 3). The overall prevalence of PAD in patients with left main coronary lesion was 61.5%. Alternatively, patients with multivessel CAD had a 2-fold higher risk of being diagnosed as having previously unrecognized PAD compared with those with single-vessel CAD (adjusted OR 2.02, 95% CI: 1.03–3.98). Moreover, patients with multivessel CAD were more likely to present with moderate to severe PAD (ankle-brachial pressure index [ABI] < 0.7) than patients with single-vessel CAD (7.3% vs 2.8%, *P* = .006). Table 2 shows the distribution of ABI values in both lower limbs and their relation to the severity of PAD. Of the ABI recordings, 12% were abnormal on the left side and 8.6% were abnormal on the right side. The severity of PAD was more prevalent in the left lower limb (right ABPI, 0.74 ± 0.15; left ABPI, 0.69 ± 0.16). The prevalence of bilateral PAD was higher in patients with multivessel CAD than in those with single-vessel disease (6.3% vs 2.1%, *P* < .001). Moreover, patients with multivessel CAD had a higher prevalence of moderate to severe PAD (ABI < 0.7) than those with single-vessel CAD (6.5% vs 2.6%, *P* < .01). In the logistic regression analysis, the factors independently associated with a greater risk of having PAD were age (OR 1.081, 95% CI: 1.053–1.109, *P* < .001), hypertension (OR 3.122, 95% CI: 1.474–5.678, *P* = .004), diabetes (OR 1.827, 95% CI: 0.975–2.171, *P* = .04), smoking (OR 1.301, 95% CI: 0.725–2.076, *P* < .001), previous CABG (OR 2.939, 95% CI: 1.385–5.219, *P* = .004), previous cerebrovascular accident (OR 3.212, 95% CI: 1.872–9.658, *P* = .003), left main CAD (OR 9.535, 95% CI: 3.978–20.230, *P* = .002), and multivessel CAD (OR 1.869, 95% CI: 1.018–2.798, *P* = .03). By using multivariate analysis, we confirmed that age (OR 1.090, 95% CI: 1.062–1.126, *P* < .001), hypertension (OR 2.357, 95% CI: 1.362–3.352, *P* = .002), diabetes mellitus (OR 1.650, 95% CI: 0.864–2.435, *P* = .045), smoking history (OR 2.228, 95% CI: 1.384–3.269, *P* = .015), previous CABG (OR 1.090, 95% CI: 1.062–1.126, *P* < .001), previous cerebrovascular accident history (OR 2.084, 95% CI: 1.295–4.116, *P* = .019), left main CAD (OR 5.114, 95% CI: 2.683–7.275, *P* = .001), and multivessel CAD (OR 1.248, 95% CI: 0.894–1.858, *P* = .04) were all independently associated with PAD (Table 3).

**Table 1:**
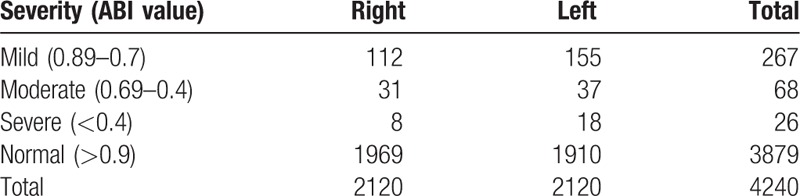
Clinical characteristic of patients with or without PAD.

**Table 2:**
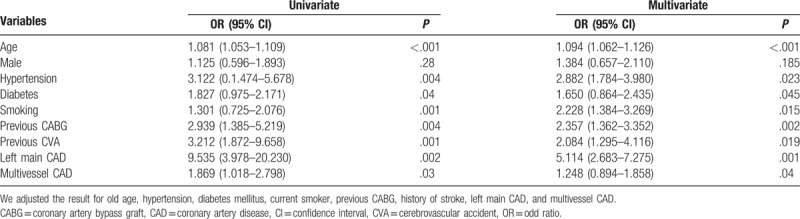
The distribution of ankle brachial index (ABI) values in both lower limbs and their relation to severity of peripheral arterial disease.

**Table 3:**
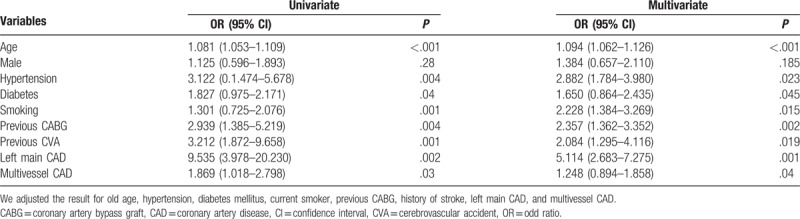
Predictors of peripheral artery disease in patients with CAD.

## 4 Discussion

This is one of the first studies in the Arabian Middle Eastern region to investigate the prevalence and predictors of asymptomatic PAD among patients undergoing coronary angiography. The study demonstrated an overall prevalence of asymptomatic PAD among patients with and without CAD of 14.7% and 4.5%, respectively. Although the patients were under the care of cardiologists, this significant finding was previously unknown. The combination of a lack of awareness among physicians and a lack of symptoms in patients led to the failure of diagnosing PAD. This high prevalence of asymptomatic PAD in our population is comparable to the prevalence reported in other Arab and non-Arab populations, such as the cohorts in Germany, Switzerland, France, Japan, and Turkey.^[21–24]^ Likewise, in the Arabian Gulf, Kownator et al. reported a prevalence of asymptomatic PAD of 13.7% in patients with a single previous coronary or cerebrovascular event, with the highest reported in Kuwait (16.3%) and the United Arab Emirates (14.7%), and the lowest in Qatar (5.3%).^[23]^ In the present study, we found that old age, hypertension, diabetes mellitus, hyperlipidemia, current smoking, previous CABG, and history of stroke were the main predictors of PAD in patients with CAD. When analyzing age in our study population, the prevalence was 11% in patients younger than 50 years. The prevalence increased to 13% in the age group 50 to 64 years and was 21% in the age group >65 years. These findings are consistent with the results of similar studies.^[25–27]^ Most of our study participants were males (74.7%), whereas females comprised only 25.3%. The occurrence of PAD was 15.0% among males and 12.5% among females, with no statistical significance (*P* = .395). Some studies showed a similar incidence of PAD, with the prevalence in men being slightly higher than that in women, whereas other studies showed that women were more likely to develop PAD than men.^[28–31]^ In our study, PAD occurred on the left side in 12% and on the right side in 8.6%. Other studies also showed a unilateral predisposition of the disease to the right or left side, as in our study.^[32,33]^ no obvious reasons for this predisposition, however using right more than left leg may decrease the process of atherosclerosis on the right side. A prior report found that smoking, hypertension, and history of cerebrovascular disease were predictors of PAD.^[34,35]^ Our results suggested that there were no significant differences in the prevalence of PAD according to the patients’ clinical presentation of either SIHD or ACS. These findings are similar to those of another report.^[34]^ In our analysis, patients with PAD had a more severe form of CAD manifested by a higher frequency of left main and multivessel coronary disease. This finding is consistent with previous reports, suggesting a greater burden of atherosclerotic disease and a later presentation, and may provide a possible explanation for the worse outcome of patients with concomitant CAD and PAD than those without PAD.^[36–38]^ Therefore, making a diagnosis of PAD in a patient with CAD should prompt the clinician to be more aggressive with risk factor intervention and to have a higher clinical index of suspicion for possible PAD symptoms. Furthermore, those patients should be considered an exceptionally high-risk group.

### 4.1 Limitations

This study has several limitations. First, patients with ischemic heart disease, whom we included, share high-risk demographic and clinical profiles known to be associated with a high prevalence of PAD. Consequently, this may have biased the study results toward a higher prevalence of PAD. Second, this is a purely noninvasive study of PAD based on ABI measurement only, without lower limb angiogram; therefore, it cannot accurately reflect the real extent and burden of underlying atherosclerosis. Third, this study does not provide information about the outcome of patients with CAD according to the presence or absence of PAD. However, previous studies have clearly demonstrated the detrimental impact of PAD on patient survival, particularly for those with preexisting CAD.

### 4.2 Clinical implications

In this study, we found that 1 of 6 patients with CAD has PAD, which was not previously known despite specialist cardiovascular care. The incidence of overlooked PAD in patients with CAD increases in frequency with advanced age and in the presence of other traditional cardiovascular risk factors. Furthermore, the presence of PAD in this population is associated with a more severe form of CAD. We recommend that all patients with CAD should be systematically screened for PAD with non-invasive ABI-based procedure.

## 5 Conclusion

The study demonstrated that the prevalence of asymptomatic PAD in patients with CAD was 14.7% and was strongly associated with higher incidence of cardiovascular risk factors, multivessel disease, and left main disease. The high prevalence of PAD in patients with CAD confirms the importance of active screening for PAD by using ABI. Our study suggested that routine determination of ABI in the clinical evaluation of all patients with CAD may help identify high-risk patients. We emphasize that clinicians should keep in mind that patients with CAD would have other atherosclerotic vascular manifestations such as PAD.

## Acknowledgments

The authors would like to acknowledge the contribution of the cardiac catheterization staff at the University of Jordan Hospital. We thank the patients, physicians, and nurses.

## Author contributions

**Conceptualization:** Akram Saleh.

**Data curation:** Akram Saleh, Hanna Makhamreh, Tareq Qoussoos, Izzat Alawwa, Moath Alsmady, Ali Shakhatreh, Lewa Alhazaymeh, Zaid A. Salah, Mohammed Jabber.

**Formal analysis:** Tareq Qoussoos, Izzat Alawwa, Moath Alsmady.

**Funding acquisition:** Akram Saleh.

**Investigation:** Akram Saleh, Hanna Makhamreh, Tareq Qoussoos, Izzat Alawwa, Moath Alsmady, Ali Shakhatreh, Lewa Alhazaymeh, Zaid A. Salah, Mohammed Jabber.

**Methodology:** Akram Saleh, Hanna Makhamreh, Tareq Qoussoos, Izzat Alawwa, Moath Alsmady, Ali Shakhatreh, Lewa Alhazaymeh, Mohammed Jabber.

**Project administration:** Akram Saleh.

**Resources:** Akram Saleh, Tareq Qoussoos, Ali Shakhatreh, Lewa Alhazaymeh, Zaid A. Salah, Mohammed Jabber.

**Software:** Akram Saleh, Hanna Makhamreh, Izzat Alawwa, Moath Alsmady, Zaid A. Salah.

**Supervision:** Akram Saleh, Hanna Makhamreh, Tareq Qoussoos, Moath Alsmady, Lewa Alhazaymeh.

**Validation:** Akram Saleh, Izzat Alawwa.

**Visualization:** Akram Saleh, Lewa Alhazaymeh, Zaid A. Salah, Mohammed Jabber.

**Writing – original draft:** Akram Saleh, Tareq Qoussoos, Izzat Alawwa.

**Writing – review & editing:** Akram Saleh, Hanna Makhamreh, Tareq Qoussoos, Izzat Alawwa, Moath Alsmady.
